# Snapshot 3D image projection using a diffractive decoder

**DOI:** 10.1038/s41377-026-02378-3

**Published:** 2026-06-10

**Authors:** Çağatay Işıl, Alexander Chen, Yuhang Li, F. Onuralp Ardic, Shiqi Chen, Che-Yung Shen, Aydogan Ozcan

**Affiliations:** 1 Electrical and Computer Engineering Department, University of California, Los Angeles, CA USA; 2 Bioengineering Department, University of California, Los Angeles, CA USA; 3 California NanoSystems Institute (CNSI), University of California, Los Angeles, CA USA

**Keywords:** Applied optics, Displays, Imaging and sensing

## Abstract

3D image display is essential for next-generation volumetric imaging; however, dense depth multiplexing for 3D image projection remains challenging because diffraction-induced cross-talk rapidly increases as the axial image planes get closer. Here, we introduce a 3D display system comprising a digital encoder and a diffractive decoder, which simultaneously projects different images onto multiple target axial planes with high axial resolution. By leveraging multi-layer diffractive wavefront decoding and deep learning-based end-to-end optimization, the system achieves high-fidelity depth-resolved 3D image projection in a snapshot, enabling axial plane separations on the order of a wavelength. The digital encoder leverages a Fourier encoder network to capture multi-scale spatial and frequency-domain features from input images, integrates axial position encoding, and generates a unified phase representation that simultaneously encodes all images to be axially projected in a single snapshot through a jointly-optimized diffractive decoder. We characterized the impact of diffractive decoder depth, output diffraction efficiency, spatial light modulator resolution, and axial encoding density, revealing trade-offs that govern axial separation and 3D image projection quality. We further demonstrated the capability to display volumetric images containing 28 axial slices, as well as the ability to dynamically reconfigure the axial locations of the image planes, performed on demand. Finally, we experimentally validated a two-plane optical prototype using a single-layer physical decoder, demonstrating close agreement between the measured results and the target images. These results establish the diffractive 3D display system as a compact and scalable framework for depth-resolved snapshot 3D image projection, with potential applications in holographic displays, AR/VR interfaces, and volumetric optical computing.

## Abstract (web summary)

A joint digital encoder and diffractive optical decoder enable snapshot 3D projection with high axial resolution, reduced cross-talk, and scalable volumetric display across closely spaced image planes.

## Graphical abstract

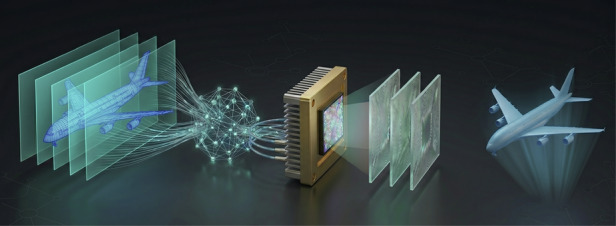

## Introduction

Three-dimensional (3D) display has emerged as a foundational technology for next-generation holography, immersive visualization, and volumetric interfaces in AR/VR systems^1–5^. By delivering accurate focal cues across depth, 3D displays can provide more natural focus accommodation and alleviate vergence–accommodation conflict compared with conventional 2D screens, thereby improving depth perception and visual comfort. To achieve high-fidelity 3D volumetric display, a diverse range of paradigms has been explored, spanning volumetric displays based on microbubble voxels^6^, acoustic trapping^7^, or photophoretic optical trapping^8^, light-field architectures such as lenticular lenslets and nanophotonic arrays^9^, as well as holographic displays that directly encode wavefronts to synthesize objects in free space^10^. Among these, holographic displays have been widely investigated due to their inherent ability to display high-resolution 3D images by providing phase and amplitude control of the optical field^11–14^. However, achieving high axial resolution across multiple closely spaced depth planes remains a challenging task. As the axial image plane spacing decreases, diffraction-induced inter-plane coupling causes severe cross-talk, degrading depth selectivity and display fidelity^15^. These limitations stem from the insufficient degrees of freedom (DoF) available in conventional single-plane modulators, which cannot simultaneously satisfy the complex field requirements of multiple axial depths^10^. Consequently, practical holographic displays are bound by fundamental trade-offs between axial resolution, signal-to-noise ratio, and the space-bandwidth product of the system.

A wide range of computational holography algorithms has been developed to synthesize volumetric holograms with improved accuracy and color performance, forming an important algorithmic foundation of modern digital holographic displays^11,16–19^. More recently, learning-based methods have been incorporated into holographic and 3D display pipelines to enable data-driven wavefront generation, phase optimization, and end-to-end co-design, often improving display quality and computational efficiency^20–23^. While computational holography algorithms have significantly improved volumetric wavefront generation, their performance remains strictly governed by the hardware-limited DoF of spatial light modulators (SLMs). Recent optical augmentation strategies have introduced additional diffractive transformations to expand the effective DoF of the optical system, including active control of volume speckle fields and learned diffractive optics that extend system capability beyond an SLM-only pipeline^24–26^.

Here, we present a snapshot 3D display system that integrates a digital encoder jointly optimized with a passive diffractive decoder composed of trainable phase layers. Within this framework, a learned diffractive decoder is co-designed with digital hologram synthesis through an encoder neural network to improve depth-dependent field shaping for snapshot 3D image projection over a desired volume. The digital encoder utilizes a Fourier-based network to extract multi-scale spatial and frequency features from input images, incorporates axial position information, and produces a single-phase representation that simultaneously encodes all input images for one-shot axial projection using a jointly optimized diffractive decoder. This end-to-end optimization, utilizing a diffractive decoder architecture, enables multi-plane snapshot 3D image projection, covering never-seen objects, with wavelength-scale axial separation between image planes while significantly suppressing inter-plane leakage/cross-talk compared to free-space-based image projection systems without a decoder. Stated differently, by embedding learned phase transformations into passive diffractive layers that serve as an optical decoder, the system physically performs depth-dependent field shaping during light propagation. This snapshot image projection architecture enables the optical routing of input images to specific depths while intrinsically suppressing inter-plane cross-talk, thereby moving beyond the inherent limitations of standard holographic image projection architectures.

We first numerically demonstrated this multi-plane snapshot image projection capability using four image planes with fine axial separation and further established the system’s scalability through simulations of volumetric targets, achieving high-fidelity simultaneous image projection across 28 depth slices axially separated by one wavelength. Throughout these numerical analyses, key performance factors, including the depth of the diffractive decoder, output diffraction efficiency, and encoded phase pixel count, were systematically evaluated to provide practical design guidelines for snapshot diffractive 3D displays. Furthermore, we investigated training strategies that enabled the dynamic adjustment of the axial position of the image projection plane across a continuous depth range rather than being restricted to fixed image planes at the output volume. Finally, we experimentally validated our snapshot image projection approach using a two-plane optical prototype operating at the visible part of the spectrum, where the measured intensity patterns closely matched our numerical simulations and target projections. Together, these results established a compact and scalable platform for high-axial-resolution snapshot 3D image display, advancing volumetric visualization and opening new opportunities for future holographic display technologies.

## Results

**Fig. 1:**
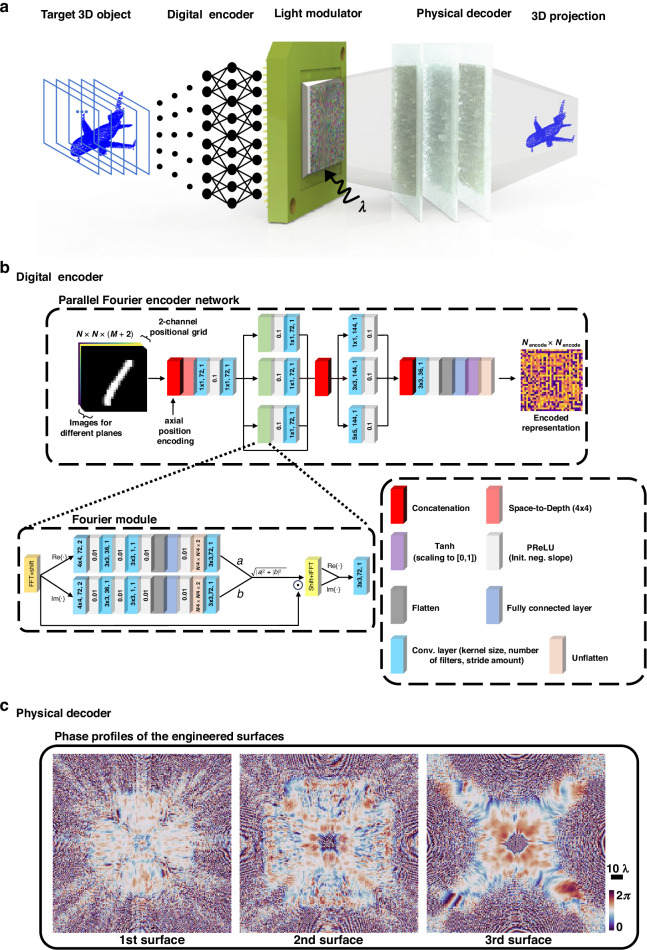
Schematic of the diffractive snapshot 3D display architecture. **a** Conceptual illustration of the diffractive snapshot 3D display system, consisting of a digital encoder and a physical decoder. **b** Structure of the digital encoder. **c** Phase profiles of the optimized diffractive surfaces constituting the physical decoder

**Fig. 2:**
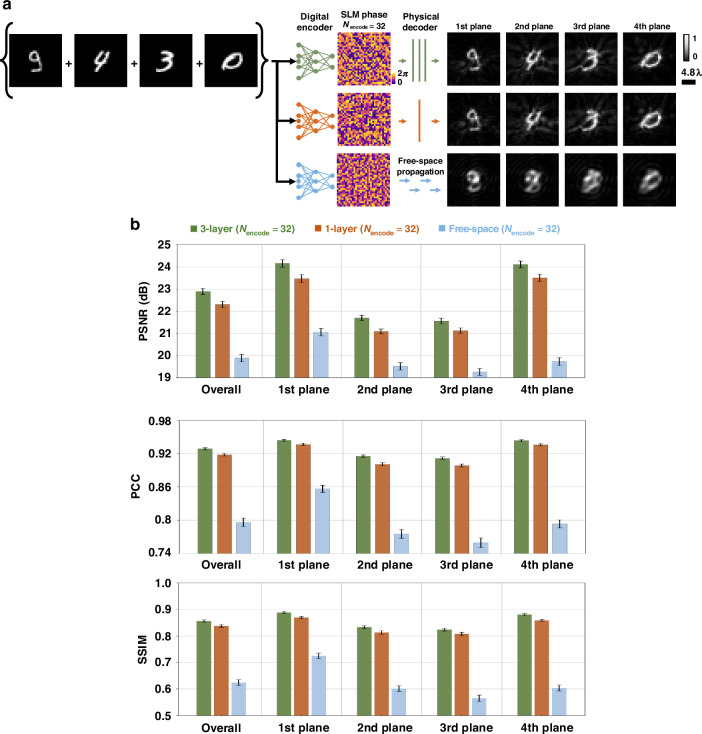
Numerical simulation results of the diffractive snapshot 3D display system. **a** Representative numerically simulated examples of snapshot multi-plane image projection, where different image content was displayed at four distinct axial planes (separated by $\Delta z=3\lambda$), reported for a three-layer diffractive decoder, a single-layer diffractive decoder, and a free-space baseline used for comparison. **b** Quantitative image display fidelity of the numerically simulated results evaluated on the test image set (never seen before) using PSNR, PCC and SSIM metrics for the three optical configurations. The multi-layer diffractive 3D display provides better image display fidelity at each projection plane

**Fig. 3:**
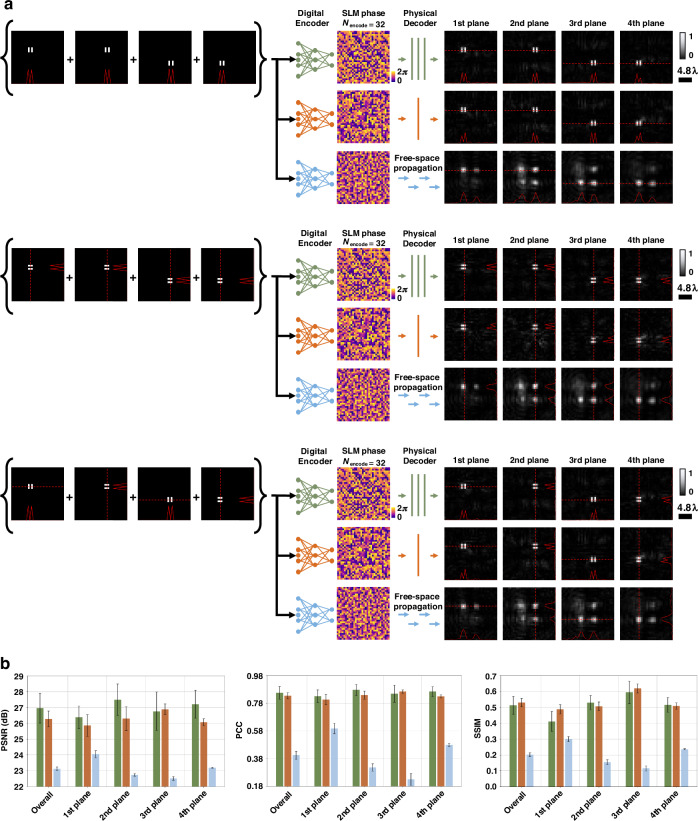
Numerically simulated multi-plane snapshot projection of line patterns using the diffractive 3D display. **a** Blind testing using vertical, horizontal, and mixed bar patterns, each with a period of 1.2*λ*. The mixed bar patterns correspond to configurations in which vertical and horizontal bars were distributed across different axial planes. These line patterns were never used during training and serve as an external test dataset. Three groups of numerically simulated images are shown, each consisting of four images corresponding to four target axial planes, for a three-layer diffractive decoder, a single-layer diffractive decoder, and the free-space baseline. The multi-layer diffractive 3D display accurately projected the line pairs at their designated axial planes, whereas the single-layer decoder configuration exhibited blur, and the free-space setup failed to display the line pairs, also exhibiting significant cross-talk among different planes. Each axial plane was separated from the neighboring planes by $\Delta z=3\lambda$. **b** Quantitative image display fidelity of the three decoding architectures on the blind-testing line-pattern image dataset as an evaluation of the external generalization of the system, measured by PSNR, PCC, and SSIM metrics

Our hybrid diffractive 3D display system consists of a digital encoder and an optical diffractive decoder that are jointly optimized. As shown in Fig. 1a, a 3D target volume was first decomposed into a stack of *M* axial slices, augmented with a 2-channel positional grid, and processed by the digital encoder, which converted the volumetric input into a phase-only representation displayed on an SLM with *N*_encode_ × *N*_encode_ pixels. This encoded light field then propagated through a sequence of spatially-optimized diffractive surfaces that formed the physical decoder, i.e., a passive optical system composed of one or more diffractive surfaces trained for decoding the encoded field into the desired intensity distributions at its designated output volume by appropriately shaping the output wavefront. The digital encoder architecture is detailed in Fig. 1b, where a parallel Fourier encoder network^27–29^ extracts multi-scale spatial and frequency-domain features from each plane, incorporates axial position encoding, and outputs an encoded phase representation that simultaneously represents all the desired images to be axially projected in a snapshot. Once trained, the diffractive decoder remains fixed as a static physical backend, while different target scenes are rendered by updating only the encoder-generated input phase pattern displayed on the SLM. This fixed decoder supports both internal generalization to unseen handwritten digit samples from the training data distribution (Fig. 2) and external generalization to previously unseen pattern classes outside the training distribution, such as the grating image dataset never seen before (Fig. 3).

We first numerically demonstrate the 3D image projection capability of the presented diffractive display system. A digital encoder and a three-layer diffractive decoder were jointly optimized end-to-end to project test input images (never seen before) onto four discrete axial planes, as shown in the first row of Fig. 2a. The phase-modulating SLM plane contained *N*_encode_ × *N*_encode_ pixels, with *N*_encode_ = 32, while the output planes were also sampled with 32 × 32 pixels, while each diffractive layer comprises 256 × 256 diffractive features, with the optimized (and static) phase profiles shown in Fig. 1c. Unless otherwise specified, the lateral pixel pitch throughout this paper was set to *d =* 0.6*λ*. The axial spacing from the SLM to the first diffractive layer, between successive diffractive layers, and from the last diffractive layer to the first output plane was set to 30*λ*. The four output image planes were uniformly separated by an axial distance of $\Delta z=3\lambda$. The display results exhibit clear image projection at different axial image planes: each randomly selected handwritten digit (forming the input test images, which have never been seen before) is accurately displayed at its assigned axial distance while being effectively suppressed at the remaining image planes, confirming accurate image projection with minimal cross-talk across different image depths.

To investigate the impact of the number of diffractive layers used, we further trained two additional architectures for comparison: a single-layer diffractive decoder and a free-space-based baseline without a diffractive decoder. For each configuration, the digital encoder and the diffractive layers (where applicable) were jointly optimized^30–32^. Overall, the three-layer diffractive design achieved the highest image display fidelity, while the single-layer configuration produced distinguishable images with noticeable degradation. In contrast, the free-space baseline without a decoder failed to form well-resolved multi-plane image projections. We further quantitatively assessed the image projection fidelity of these architectures (for 10,000 test images never seen before) using the peak signal-to-noise ratio (PSNR), the Pearson correlation coefficient (PCC) and the structural similarity index (SSIM), as summarized in Fig. 2b (see “Methods” for details). Averaged across the four output planes, the three-layer decoder achieved 22.9 dB PSNR, 0.93 PCC, and 0.86 SSIM, outperforming the single-layer decoder (22.3 dB, 0.92, 0.84) and the free-space baseline performance (19.9 dB, 0.79, 0.62). These results confirm that the three-layer decoder enhances the overall quality of 3D image projection. Notably, the improvement from one to three diffractive layers is consistent but more moderate compared with the performance gain obtained when moving from free-space propagation to a learned single-layer decoder, suggesting that the introduction of the first diffractive layer already provides substantial decoding capability, whereas additional diffractive depth further refines the image projection quality.

To further evaluate the spatial-frequency handling capability of the diffractive 3D display system, we performed blind testing using a set of tightly spaced line patterns with a period of 1.2*λ*, as shown in Fig. 3a. These grating patterns, including vertical and horizontal line-pairs as well as their combinations across axial planes, were never used during training and served as an external test dataset. The three-layer diffractive decoder successfully displayed the paired gratings at their designated axial planes, while strongly suppressing cross-talk and their leakage at other depths. In comparison, the single-layer decoder system yielded partial reconstructions with noticeable blur and axial cross-talk, whereas the free-space baseline failed to resolve the grating pairs and had major cross-talk across different image planes. These qualitative observations are further supported by the quantitative evaluation in Fig. 3b. Using the same quantitative metrics as in Fig. 2, the three-layer diffractive decoder achieved the best overall performance, followed by the single-layer decoder, while the free-space baseline yielded the lowest values across all three performance metrics. These results are consistent with the visual reconstructions in Fig. 3a and further confirm that multi-layer diffractive decoding improves the fidelity and structural accuracy of the projected images across the target axial planes.

**Fig. 4:**
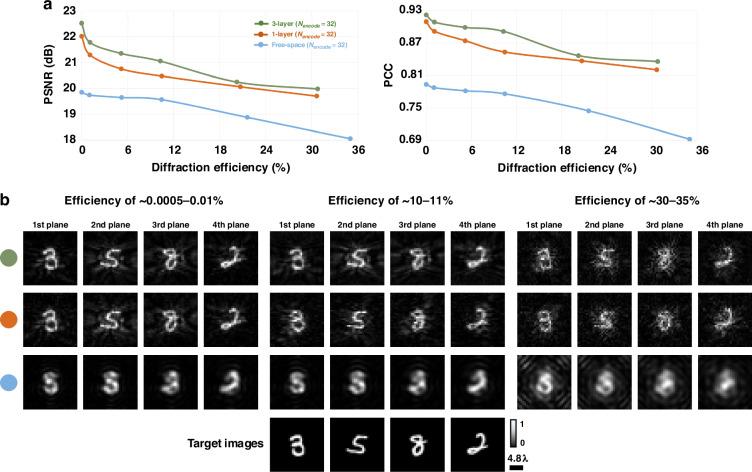
Numerically simulated diffraction efficiency vs. performance trade-off analysis of the snapshot diffractive 3D display. **a** Output PSNR and PCC values of the numerically simulated results as a function of the diffraction efficiency for three optical configurations: a three-layer diffractive decoder, a single-layer diffractive decoder, and the free-space baseline, showing an inherent efficiency–performance trade-off. **b** Representative numerically simulated multi-plane projections at low, moderate, and high diffraction efficiency levels. Low-efficiency outputs were clean, moderate efficiency provided balanced contrast, while high diffraction efficiency introduced increased speckle and inter-plane cross-talk. The colored circles on the left correspond to the same three-layer diffractive decoder, single-layer diffractive decoder, and the free-space baseline reported in (**a**). Axial separation between image planes was $\Delta z=3\lambda$

We then evaluated the performance trade-off between the output diffraction efficiency and the display fidelity of the system by introducing an energy efficiency constraint to the training loss function, while maintaining the same four-channel input configuration as in Fig. 2. These 3D display systems were optimized using a composite reconstruction error alongside a diffraction efficiency penalty term (see “Methods” for details). As shown in Fig. 4a, the PSNR and PCC values decreased as the output diffraction efficiency increased, revealing a clear efficiency–fidelity trade-off: higher output diffraction efficiency improved overall image brightness but also introduced stronger interference artifacts and axial cross-talk, which reduced display fidelity. Representative image projections at three output efficiency regimes: low (∼0.0005–0.01%), moderate (∼10–11%), and high (∼30–35%), are shown in Fig. 4b. At low diffraction efficiencies, the display results were structurally clean, whereas increasing the efficiency introduced visible speckle noise. We further compared free-space propagation, single-layer, and three-layer diffractive decoder architectures under matched efficiency regimes. Across all regimes, the three-layer decoder configuration consistently delivered the best 3D image display quality, followed by the single-layer decoder design, while the free-space propagation without the diffractive decoder yielded the lowest performance. Notably, even in the lowest diffraction efficiency regime, the free-space propagation baseline exhibited inferior performance, with strong cross-talk observed between axial planes, underscoring the fundamental advantages of increased depth of the diffractive decoder.

**Fig. 5:**
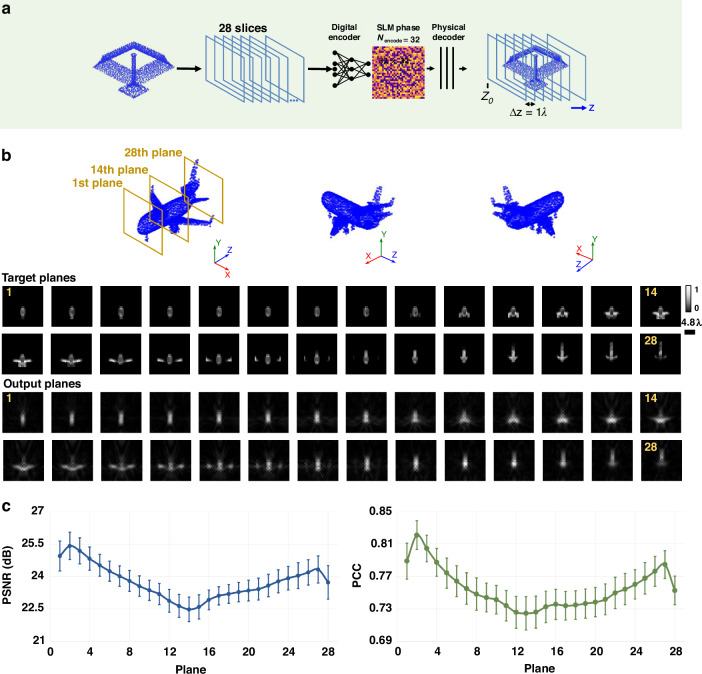
Numerically simulated volumetric multi-slice projection using the snapshot diffractive 3D display. **a** Schematic of the 28-slice volumetric input and projection process using a digital encoder and a diffractive decoder, where a stack of axial slices was encoded into a single phase pattern and projected in a snapshot with a uniform axial spacing of $\Delta z=1\lambda$. **b** Target slices and corresponding numerically simulated projected outputs at selected axial positions. **c** PSNR and PCC values of the numerically simulated results were evaluated across all 28 axial planes, each with an axial separation of $\Delta z=1\lambda$

Building on the preceding analysis, which focused on multi-plane image projection for a few discrete axial planes, we extended our method to a continuous volumetric image projection scenario involving a substantially larger number of axial slices. We selected a 28-slice setting as a representative dense volumetric workload to move well beyond the four-plane projection benchmark and to stress-test the scalability of the framework under heavier axial multiplexing, while keeping the slice-by-slice projection results computationally tractable and visually interpretable. To accommodate the increased axial density, we trained a new model specifically optimized for this higher-resolution volumetric image projection task (see “Methods” for details). Fig. 5a reports the diffractive 3D display capability of our system using a 28-slice airplane model (a test object never seen before), which imposed a higher axial workload on the system. The 3D volume was decomposed into 28 axial slices with a uniform separation of $\Delta z=1\lambda$, and these axial slices were encoded to a single-phase pattern for simultaneous projection in a snapshot. During inference, this single encoded hologram reconstructed each slice at its assigned axial depth through the optimized diffractive decoder, enabling volumetric projection across an extended axial range. As illustrated in Fig. 5b, the system successfully reproduced the main structural components of the ‘airplane’ across all 28 axial planes. Slightly reduced fidelity was observed near the central slices, whereas the outer slices exhibited visually clearer results. This trend is likely caused by stronger inter-plane cross-talk in the middle of the displayed volume, where each slice is influenced by neighboring target planes on both sides, while the boundary slices are affected predominantly from one side only. This effect can be further aggravated by increased geometric complexity of the projected patterns, which places a greater demand on the accurate formation and axial separation of the target wavefronts. As a result, in the 28-plane test case, where both the volumetric density and pattern complexity are higher, the reduction in image projection fidelity near the central slices becomes more noticeable. Quantitative metrics in Fig. 5c further confirmed this trend: both PSNR and PCC values showed a dip near the mid-slices, followed by improvement toward the volume boundaries. Together, these results demonstrate that the proposed snapshot 3D diffractive display can scale from a few-plane image projection to large volumetric workloads, supporting multi-slice 3D rendering in a snapshot using a single input phase encoding.

**Fig. 6:**
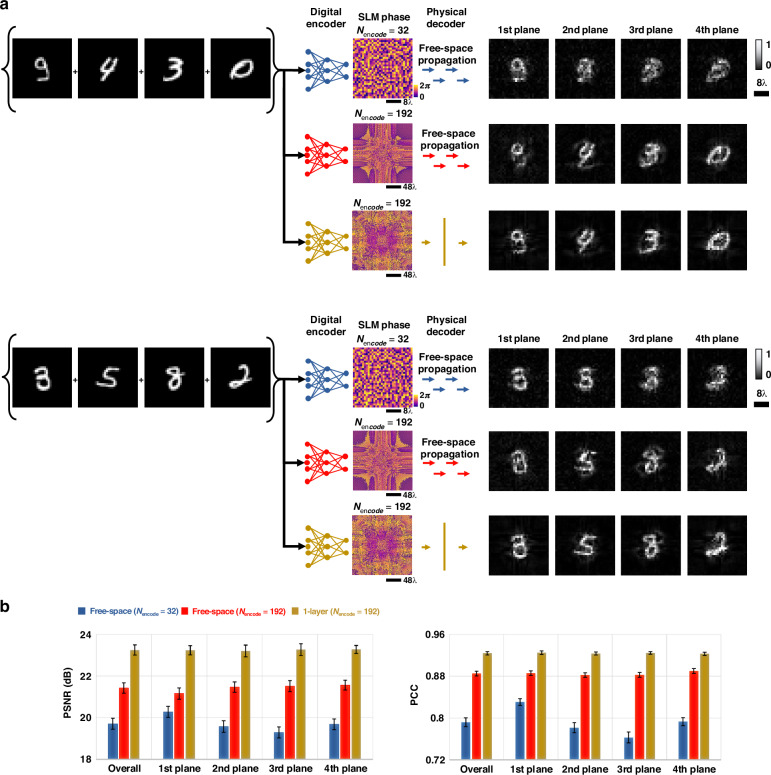
Numerical simulations showing the impact of the number of pixels used for the encoder phase pattern. **a** Representative numerically simulated multi-plane snapshot image projection examples obtained using two sampling resolutions, denoted as *N*_*encode*_ *× N*_*encode*_ with ${N}_{{encode}}\in \{32,\,192\}$ (i.e., 32 × 32 and 192 × 192), evaluated under free-space propagation and a single-layer diffractive decoder. Increasing the number of encoder pixels increases the effective space–bandwidth product of the encoded wavefront, enabling a larger usable field of view, improved visual clarity, and reduced inter-plane cross-talk. **b** PSNR and PCC values of the numerically simulated results evaluated across all axial planes as a function of *N*_*encode*_, showing consistent fidelity improvements with increased pixel count and superior performance of the diffractive decoder compared to free-space propagation

To further dissect the factors affecting diffractive 3D display quality, we examined the interplay between the number of encoder pixels and the presence of a learned diffractive decoder. Specifically, we compared two SLM configurations parameterized as *N*_encode_ × *N*_encode_, with ${N}_{{\rm{encode}}}\in \{32,\,192\}$, under two propagation conditions: free-space-based propagation and a single-layer diffractive decoder. In these analyses, the pixel pitch was fixed to $d=1\lambda$. As shown in Fig. 6a, increasing the number of encoder pixels ${N}_{{\rm{SLM}}}{=N}_{{\rm{encode}}}^{2}$ markedly improved the perceptual sharpness of the test images (never seen before) under free-space propagation; however, these reconstructions still exhibited pronounced inter-plane leakage due to limited modulation capability. Coupling the higher DoF phase pattern $\left({N}_{{\rm{encode}}}=192\right)$ with a single diffractive layer-based decoder (256 × 256 features) yields a substantial improvement in 3D image projection fidelity. As shown in Fig. 6b, the overall PSNR increases from 19.8 dB (${N}_{{\rm{encode}}}=32$, free-space) to 21.4 dB (${N}_{{\rm{encode}}}=192$, free-space) and reaches 23.2 dB with the diffractive decoder, while the PCC metrics improve from 0.79 to 0.88 and 0.92, respectively. These results indicate that higher-resolution phase modulation alone is insufficient for depth-multiplexed 3D image display; they also underscore the critical role of an optimized diffractive decoder, which is essential for suppressing axial cross-talk and decoupling adjacent axial planes from each other, thereby enabling a high-fidelity 3D display that higher-resolution phase modulation alone cannot achieve.

**Fig. 7:**
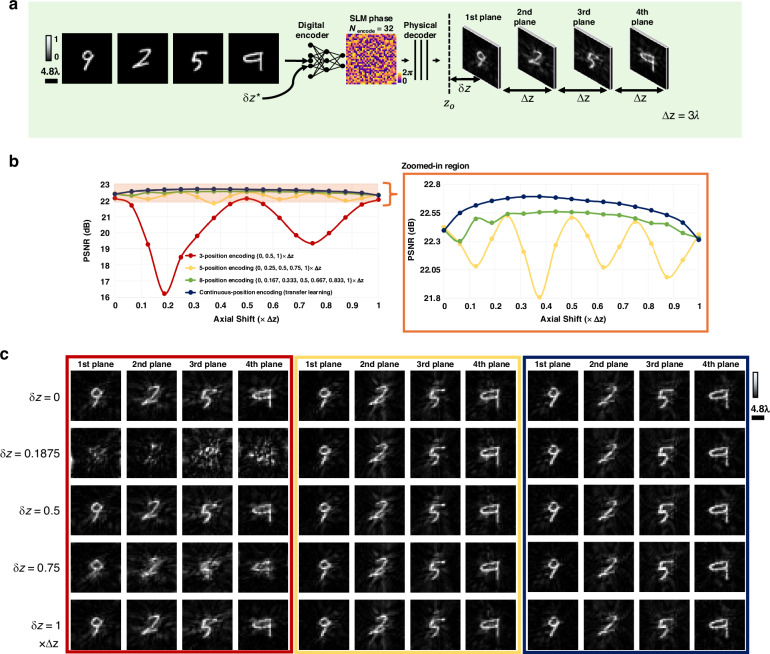
Numerically simulated axial-shift adjustment ability of the snapshot diffractive 3D display under different axial encoding strategies. **a** Schematic of the distance adaptive diffractive 3D display, where an axial shift $\delta {z}^{* }$ was fed into the digital encoder to adjust the starting axial position of the projected display stack. **b** Output PSNR of the numerically simulated results as a function of the input-controlled axial shift *δz* during testing for models trained with 3-position, 5-position, 8-position, and continuous-position encoding. Denser axial sampling during the training produced smoother PSNR curves as a function of image depth. **c** Representative numerically simulated multi-plane snapshot image projections at different values of *δz*

We further assessed the ability of the diffractive 3D display to dynamically tune the target projection plane. To this end, we varied the target axial offsets $\delta {z}^{* }$ used to condition the digital encoder, as shown in Fig. 7a. This strategy enables the encoder digital network to dynamically generate phase patterns for different desired axial locations. During training, we considered four axial sampling strategies over $[0,\,\Delta {\mathrm{z}}]$: discrete sampling at 3, 5, or 8 uniformly spaced axial planes, and continuous sampling where $\delta {z}^{* }\sim U(0,\Delta z)$, where $\Delta z=3\lambda$. The continuous axial sampling model was initialized from the 8-position model via transfer learning to accelerate its convergence. We blindly tested all models over a continuous sweep of axial shifts, $\delta z/\Delta z\in [\mathrm{0,1}]$, which was used as an input to the encoder network to set the desired axial projection distance, as shown in Fig. 7b. Sparse axial encoding (3 or 5 axial positions) led to pronounced, oscillatory behavior in the output PSNR, with sharp degradations at intermediate depths that were not used during training. In contrast, denser discrete sampling and continuous conditioning of the digital encoder yielded smooth, consistent performance across the entire axial range, thereby improving the overall 3D image display fidelity. Representative results in Fig. 7c corroborate these trends: sparsely trained models exhibit blur and geometric distortions at intermediate axial offsets, whereas the continuously trained model maintains sharp, structurally stable projections throughout $[0,\,\Delta {\mathrm{z}}]$. Collectively, these results indicate that denser axial sampling during training is crucial for empowering the system with robust axial position adjustment capabilities, while also improving overall image display performance.

**Fig. 8:**
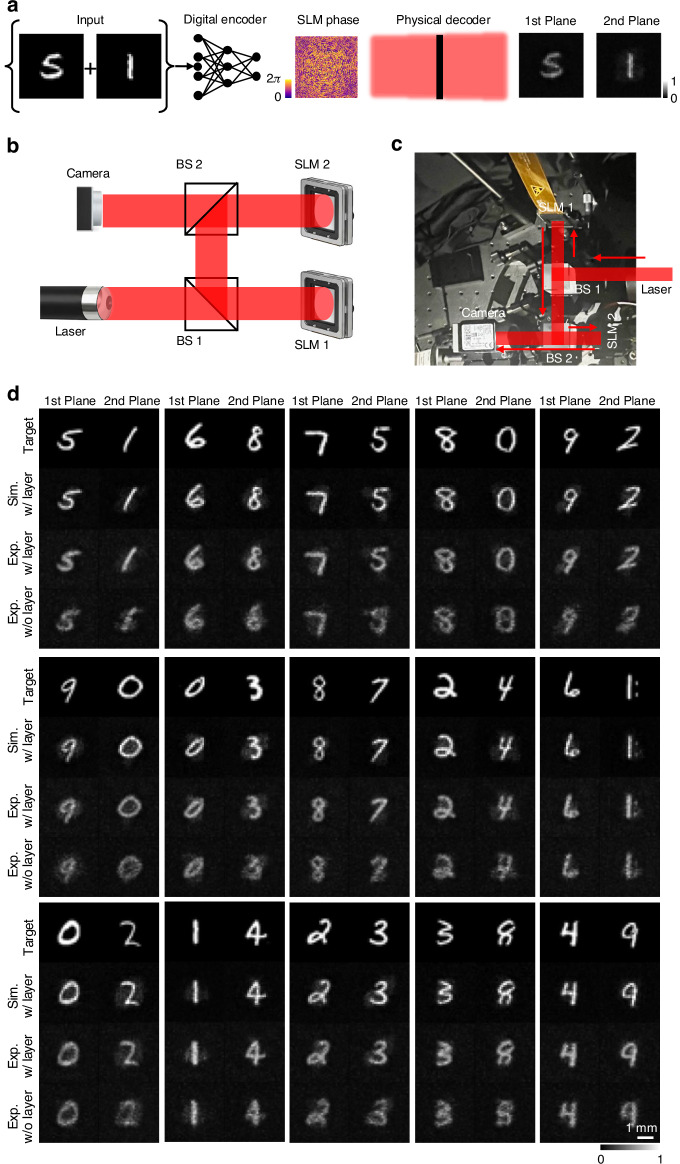
Experimental validation of the diffractive snapshot 3D display system. **a** Schematic of the two-plane projection setup. **b** Optical layout of the experimental system. **c** Photograph of the physical setup showing the laser source, beam splitters, two reflective SLMs, and the imaging camera. **d** Experimental results for multiple input test objects (never seen before). For each sample, target images (Target), simulated results with the diffractive layer (Sim. w/ layer), experimentally measured results with the diffractive layer (Exp. w/ layer), and experimentally measured free-space baseline without the diffractive layer (Exp. w/o layer) are shown at the 1st and 2nd planes (Δ*z* = 1 cm). BS: Beam Splitter, SLM: Spatial Light Modulator. Also see Fig. S1 for additional experimental results that further support the same conclusions

Beyond these numerical analyses, we also experimentally validated the presented diffractive 3D display system. We jointly trained a digital encoder with a single-layer physical decoder to simultaneously project test images (never seen before) onto two axial image planes, as illustrated in Fig. 8a. In this configuration, two test images were first processed by the digital encoder to generate a phase-only pattern, which was then displayed on SLM1. As shown in the schematic in Fig. 8b, coherent illumination (*λ =* 650 nm) interacted with this phase modulation, propagated through the optical path, and was subsequently decoded by SLM2, implementing the physical decoder function by displaying the learned, static phase profile of the diffractive layer. A photograph of the experimental setup is provided in Fig. 8c. Fig. 8d visualizes the target images, numerical simulations, and experimentally captured results at the 1st and 2nd axial planes, separated by Δz = 1 cm. The experimental outputs closely match the corresponding simulations and target patterns across a diverse set of handwritten digits (never seen before), whereas the free-space baseline exhibits lower fidelity. These results demonstrate that the trained hybrid (digital–analog) architecture with an optimized diffractive decoder was accurately realized in the visible spectrum and improves the projection fidelity beyond free-space propagation alone. These results further confirm the robustness of the learned design and the physical decoder’s ability to faithfully reproduce multi-plane image projections. Additional experimental results are also provided in Fig. S1, further supporting the same conclusions.

## Discussion

In this work, we introduced a hybrid 3D diffractive display system that jointly optimizes digital hologram synthesis and multi-layer diffractive decoding to improve axial plane separability in depth-multiplexed 3D image projection in a snapshot. By training a digital encoder together with an optical diffractive decoder, the system learned to simultaneously route different test images to distinct axial planes and suppress their leakage across depth. Our results demonstrate that the multi-layer configuration consistently outperforms single-layer and free-space baselines, showing that diffractive depth is essential for accurate axial routing and suppression of inter-plane leakage, in the sense that additional controllable degrees of freedom during propagation can be used to route energy to designated axial planes that are densely packed. The framework also generalizes beyond the training distribution, successfully displaying, for example, unseen high-frequency 3D patterns. Additionally, we demonstrated the scalability of this approach to larger volumetric workloads by displaying a 28-slice volumetric scene with an axial separation of $\Delta z=1\lambda$ between two successive image planes.

Our ablation studies also provide practical design guidance for hybrid diffractive 3D displays. We systematically investigated key factors that govern system performance, including diffraction efficiency, the number of encoder pixels used for phase display, and the ability to dynamically adjust the target projection planes. An explicit trade-off between the output diffraction efficiency and 3D image display fidelity was observed, where at higher diffraction efficiencies, we observed stronger artifacts and increased cross-talk. We also showed that while increasing the encoder pixel counts controlled by the encoder network improved image sharpness, it could not eliminate depth leakage on its own; incorporating a learned diffractive decoder (jointly optimized with the encoder network) provided significant gains beyond the high-resolution free-space case, indicating that the optical decoder contributed a complementary and essential depth-selective snapshot 3D image projection capacity. Regarding depth-tunable 3D image displays, depth conditioning of the encoder enabled the dynamic adjustment of target image planes; furthermore, dense axial sampling during training was found to be crucial for empowering the system with robust image depth adjustment capabilities and ensuring smooth performance across intermediate image depths. Taken together, our results underscored the importance of joint digital–optical optimization, where computational encoding and physical wavefront shaping operated in synergy to maximize the effective space–bandwidth product of the overall snapshot 3D image projection system. Finally, we experimentally validated a two-plane implementation of the proposed framework using a single-layer physical decoder, where the measured images closely matched the simulations and successfully reproduced the target images, confirming the feasibility of the hybrid diffractive system for snapshot 3D image display. In our analyses, an important general observation is that the outer planes within the projection volume consistently exhibited slightly better image fidelity than the central image planes. This trend likely arises from the finite depth-sectioning capability of the current architecture: central planes are bounded by target slices on both sides, making them inherently more susceptible to inter-plane cross-talk and residual out-of-plane leakage, whereas boundary slices at the edges of the projection volume suffer from cross-talk predominantly from a single adjacent image plane. This behavior should not be interpreted as an intrinsic limitation of diffractive volumetric display in general, but rather as a manifestation of the limited axial resolution under densely multiplexed multi-plane image projection. In principle, this cross-talk can be mitigated by either increasing the axial spacing between the target planes or expanding the available optical degrees of freedom (e.g., by using deeper and wider diffractive decoders with a higher numerical aperture, NA).

We should note that the functionality of the multi-layer diffractive decoder cannot be approximated by a more complex, purely digital module concatenated after the electronic encoder, with its output subsequently loaded onto a light-modulation device, e.g., an SLM. In such an architecture, from the SLM plane to the observation volume of the projected images, the optical system is solely governed by a spatially invariant complex-valued convolution operation acting on the diffracting field from the SLM plane onto the image volume; such an optical transformation cannot represent the spatially varying complex-valued point spread functions^33–38^ that an optimized diffractive decoder in general represents. Therefore, mathematically, a spatially optimized optical diffractive decoder positioned after the SLM forms a superset decoder architecture compared with free-space-based convolutional decoders. Moreover, a phase-only modulation device cannot directly reproduce the same complex-field functionality as a multi-layer diffractive structure can. By distributing trainable degrees of freedom across multiple axial planes, the diffractive decoder can jointly shape both the amplitude and phase of the output light field, providing a much richer representation than a single phase modulation SLM can. For 3D display applications intended for direct human perception, our architecture provides enhanced reconstruction fidelity without adding any active power overhead. In addition to these, implementing an additional digital computing module to enhance the functional representation of the projected phase patterns at an SLM would shift the transformation burden back to the electronic domain, significantly increasing computational complexity, latency, and energy consumption for every displayed image volume. The physical execution of the diffractive decoder involves a massive number of inter-layer connections and high-dimensional field manipulations. In a digital twin, these operations would incur substantial memory bottlenecks and power penalties; in contrast, our physical diffractive decoder performs these large-scale multiplications passively through optical diffraction and propagation, enabling parallel processing with zero operational energy consumption. Consequently, the physical diffractive decoder serves as a scalable, energy-efficient optical computing platform that overcomes the inherent speed and power limitations of purely digital systems.

The presented framework is not restricted to a specific operating wavelength, and the same hybrid digital–optical architecture can, in principle, be transferred to other spectral bands by appropriately adjusting the optical configuration. In practice, changing the wavelength can affect the system dimensions, fabrication tolerances, and the material or modulator response, which may in turn influence the realized performance. In this work, an SLM-based decoder was adopted experimentally because it provides a practical, reconfigurable platform for validating the framework across different tasks and configurations. However, the diffractive decoder can, in principle, be realized as a 3D-fabricated passive optical element rather than an SLM. In visible-wavelength-scale systems, such passive decoders may be implemented using high-resolution fabrication methods such as two-photon polymerization^39,40^, grayscale lithography^41^, electron-beam lithography, or nanoimprint-based processes, depending on the required feature size, material platform, and fabrication throughput. In addition, although only a single-layer diffractive decoder was experimentally demonstrated here, extending the decoder to multiple layers can provide additional optical degrees of freedom for 3D image projection and improved axial projection performance, at the cost of greater fabrication complexity. In particular, multi-layer realizations impose stricter requirements on fabrication fidelity and on lateral and axial alignment between layers. These challenges may be mitigated through error-aware optimization strategies^42,43^, in which fabrication tolerances, phase quantization, finite feature resolution, and layer misalignments are incorporated into the training model, including vaccination-based schemes that explicitly introduce random perturbations during training to improve hardware robustness.

While the presented diffractive 3D display system demonstrates strong volumetric projection capabilities, the trained hybrid model remains specific to the optical configuration used during optimization, including the pixel pitch, axial spacing, and output volume geometry. Accordingly, substantial changes to these system/design parameters generally require re-optimization of the encoder–decoder pair and, for static physical implementations, may also necessitate reprogramming or refabrication of the diffractive decoder layers. Additional practical challenges arise when translating the framework to more complex physical systems. In particular, realizing multi-layer diffractive decoders places an increasing demand on precise lateral and axial alignments and stability as the diffractive layer count increases; this motivates vaccination-based training strategies, where perturbations are intentionally introduced during training to improve tolerance to fabrication and alignment errors^42,43^. Extending the approach to broadband or color operation represents another direction, which would require joint optimization across wavelengths and careful consideration of material dispersion in the decoder layers^11,44^. Furthermore, scaling to higher spatial resolution or dynamic volumetric scenes would increase training and memory costs due to the requirement for larger propagation models and finer target grids, necessitating the adoption of efficient parallel computing techniques and distributed optimization strategies^45^. Consequently, continued advances in 3D fabrication of diffractive decoders, calibration procedures and in situ learning methods^46,47^, as well as model-based training and higher-resolution modulators, would be essential for translating the presented framework into practical systems. At the same time, the close agreement observed in our experimental optical prototype with respect to numerical results indicates that extending the framework toward more capable physical systems is a practical engineering challenge rather than a conceptual incompatibility between the learned design and the optical implementation.

Looking forward, the presented diffractive snapshot 3D display framework opens opportunities for compact holographic displays, multi-depth AR/VR projection, volumetric microscopy, and real-time 3D visualization systems. Its ability to encode and route information across depth suggests potential extensions to multispectral operation and multi-perspective holography. We anticipate that this hybrid approach will contribute to the development of the next generation of high-fidelity 3D display technologies.

## Materials and methods

### Digital encoder design

As illustrated in Fig. 1b, we employed a parallel Fourier encoder network as the computational front end to synthesize the snapshot wavefront needed for 3D image projection. The network took a target 3D volume, represented as an *M*-channel stack of 2D intensity slices, and output a phase-encoded single pattern that modulated the incident field to display the desired volumetric image at the specified output volume. To support depth-adaptive and spatially-aware phase encoding, we augmented the input with additional spatial context channels. First, a 2-channel normalized Cartesian coordinate grid $(x,{y})$ was appended to the input slices, allowing the translation-invariant convolutional kernels to learn accurate wavefront shaping required for 3D image projection. Second, inside the network, the target axial shift $\delta {z}^{* }$ was spatially broadcast into a depth plane map and further concatenated with the input, conditioning the network on the desired axial projection depth. The digital encoder comprises convolutional layers coupled with multiple Fourier modules that operate in parallel. This multi-branch design enabled the network to capture complementary global and local image features. The Fourier modules operated in the frequency domain, learning global filters and modeling long-range dependencies. The real and imaginary components of the frequency representation were processed separately and then recombined into real-valued feature maps. Convolutional blocks with varying kernel sizes extract robust, multi-scale local features in the spatial domain. These global and local feature maps were then concatenated and passed through a fully connected layer to produce the final encoded phase-only pattern for the snapshot 3D image projection.

### Physical decoder design

The physical decoder consists of a sequence of passive and stationary diffractive layers positioned after the encoder spatial light modulator. We modeled the system using monochromatic illumination with wavelength *λ*. The optical forward model was expressed as a sequence of free-space propagations and phase modulations. Free-space propagation between two consecutive planes separated by an axial distance ${z}_{d}$ was modeled using the angular spectrum approach:

1 $$u\left(x,\,y,\,z+{z}_{d}\right)={{\mathcal{F}}}^{-1}\left\{{\mathcal{F}}\left\{u\left(x,y,\,z\right)\right\}\cdot H({f}_{x},{f}_{y};{z}_{d})\right\}$$

where $u\left(x,{y},z\right)$ denotes the complex optical field at plane *z* along the optical axis, and $u\left(x,{y},{z}+{z}_{d}\right)$ is the resulting propagated field after the distance ${z}_{d}$. ${f}_{x}$ and ${f}_{y}$ are the spatial frequencies along the *x*- and *y*- directions, respectively. ${\mathcal{F}}$ and ${{\mathcal{F}}}^{-1}$ represent the two-dimensional Fourier and inverse Fourier transforms, and $H({f}_{x},\,{f}_{y};{z}_{d})$ is the free-space transfer function:

2 $$H\left({f}_{x},\,{f}_{y};{z}_{d}\right)=\left\{\begin{array}{rl}\exp \left\{jk{z}_{d}\sqrt{1-{\left(\frac{2\pi {f}_{x}}{k}\right)}^{2}-{\left(\frac{2\pi {f}_{y}}{k}\right)}^{2}}\right\}, & {f}_{x}^{2}+{f}_{y}^{2} < \frac{1}{{\lambda }^{2}}\\ 0, & {f}_{x}^{2}+{f}_{y}^{2}\ge \frac{1}{{\lambda }^{2}}\end{array}\right.$$

where $$j=\sqrt{-1}$$ and $$k=\frac{2\pi }{\lambda }$$.

Each diffractive layer was modeled as a thin phase-only modulation element with negligible absorption. The transmission coefficient of the ${k}^{{th}}$ layer located at $z={z}_{k}$ is:

3 $$t\left(x,y\right)=\exp \left\{j\phi \left(x,y,\,{z}_{k}\right)\right\}$$

where $\phi \left(x,y,\,{z}_{k}\right)$ is the trainable phase modulation value. These phase values constituted the learnable parameters of the 3D image projection diffractive decoder.

In the experimental implementations of the diffractive projection system, misalignments between the diffractive layers may degrade the projection quality. To enhance robustness, we introduced random misalignments during training. The lateral and axial displacements $D=({D}_{x},\,{D}_{y},\,{D}_{z})$, were introduced as independent, uniformly distributed random variables. These displacement errors ${D}_{x},\,{D}_{y},$ and ${D}_{z}$ were modeled by uniformly distributed random variables,

4 $${D}_{x} \sim U\left(-{\Delta }_{x},{\Delta }_{x}\right),{D}_{y} \sim U\left(-{\Delta }_{y},{\Delta }_{y}\right),{D}_{z} \sim U\left(-{\Delta }_{z},{\Delta }_{z}\right)$$

where ${\Delta }_{x}$, ${\Delta }_{y}$, and ${\Delta }_{z}$ represent the maximum displacements along the *x*-, *y*-, and *z*- axes, respectively. In our designs, we set the misalignment bounds uniformly as ${\Delta }_{\max }={\Delta }_{x}={\Delta }_{y}={\Delta }_{z}=0.5d,$ where *d* = 8 µm is the pixel pitch for the experimental setup.

### Training loss function and performance evaluation metrics

The 3D diffractive display was trained to simultaneously project a set of target images ${\left\{{I}_{{\rm{target}},{i}}\right\}}_{i=1}^{M}$ onto *M* designated axial planes. Given the complex output field ${E}_{{\rm{out}},{i}}(x,{y})$ at the *i*-th output plane, the projected intensity is given by:

5 $${O}_{i}(x,\,y)={\left|{E}_{{\rm{out}},\,i}(x,y)\right|}^{2}$$

Each ${O}_{i}(x,{y})$ was then normalized, with average pixel binning to obtain ${O}_{{\rm{bin}},{i}}(x,y)$, which was compared against the corresponding target intensity ${I}_{{\rm{target}},{i}}(x,y)$. The reconstruction loss was defined as a weighted combination of a mean squared error (MSE) term and a Pearson correlation coefficient (PCC) term:

6 $${L}_{{\rm{rec}}}={L}_{{\rm{MSE}}}+{L}_{{\rm{PCC}}}$$

The MSE loss was computed as:

7 $${L}_{\mathrm{MSE}}=\frac{1}{M}\mathop{\sum }\limits_{i=1}^{M}\frac{1}{{HW}}\mathop{\sum }\limits_{x,y}{\left|{O}_{\mathrm{bin},i}(x,y)-{I}_{\mathrm{target},i}(x,y)\right|}^{2}$$

where *H* and *W* denote the height and width of the images. The PCC loss was computed as:

8 $${L}_{\mathrm{PCC}}=\frac{1}{M}\mathop{\sum }\limits_{i=1}^{M}\left(1-\mathrm{PCC}\left({O}_{\mathrm{bin},i},{I}_{\mathrm{target},\,i}\right)\right)$$

with,

9 $$\mathrm{PCC}\left(O,\,I\right)=\frac{{\sum }_{x,y}\left(O-{\mu }_{O}\right)\left(I-{\mu }_{I}\right)}{\sqrt{\left({\sum }_{x,y}{\left(O-{\mu }_{O}\right)}^{2}\right)\left({\sum }_{x,\,y}{\left(I-{\mu }_{I}\right)}^{2}\right)}}$$

and ${\mu }_{O}$ and ${\mu }_{I}$ denote the spatial means of *O* and *I*, respectively. To regulate output diffraction efficiency during training, an efficiency penalty term was introduced, defined as:

10 $${L}_{{\rm{eff}}}={\rm{m}}{ax}\left(0,{\eta }_{{\rm{target}}}-{\eta }_{{\rm{batch}}}\right)$$

where ${\eta }_{{\rm{target}}}$ represents the desired diffraction efficiency threshold, and ${\eta }_{{\rm{batch}}}$ is the measured diffraction efficiency for the current mini-batch.

To further enhance axial selectivity and prevent degenerate complex field solutions during early stages of the optimization, two complex-field correlation-based regularization terms were employed. The first term penalized the similarity between complex fields at adjacent axial planes in the output volume:

11 $${L}_{\mathrm{cor}}=\frac{1}{M-1}\mathop{\sum }\limits_{i=1}^{M-1}\left[\frac{{\sum }_{x,y}{E}_{\mathrm{out},i}(x,y){E}_{\mathrm{out},i+1}^{\ast }(x,y)}{{\Vert {E}_{\mathrm{out},i}\Vert }_{2}{\Vert {E}_{\mathrm{out},i+1}\Vert }_{2}}\right]$$

where ${\Vert E\Vert }_{2}=\sqrt{{\sum }_{x,y}{|E(x,y)|}^{2}}$. The second term, shown below, enforces a low correlation between the output field ${E}_{{\rm{out}},i}$ and a synthetic reference field ${E}_{{\rm{ref}}}$ constructed using the target amplitude with an artificially designed phase profile. Specifically, the amplitude of ${E}_{{\rm{ref}}}$ is defined as the square root of the target intensity, such that the reference field shares the same amplitude distribution as the target. Its phase was generated as a weighted mixture of two components: a periodic binary phase pattern, in which adjacent columns alternated between phase values of 0 and *π*, and a random phase pattern uniformly distributed over $[\mathrm{0,2}\pi )$. The corresponding complex fields were linearly combined to form the final reference field. And the random-phase component was assigned a larger weight than the periodic component, so that ${E}_{{\rm{ref}}}$ was dominated by a spatially varying random-like phase structure while still retaining a weak periodic modulation. This design discourages outputs from resembling simple gratings or random-phase fields, and instead promotes the optimizer toward physically meaningful, task-specific wavefronts, i.e.,

12 $${L}_{\mathrm{ref}}=\frac{1}{M}\mathop{\sum }\limits_{i=1}^{M}\left[\frac{{\sum }_{x,y}{E}_{\mathrm{out},i}(x,y){E}_{\mathrm{ref}}^{\ast }(x,y)}{{\Vert {E}_{\mathrm{out},i}\Vert }_{2}{\Vert {E}_{\mathrm{ref}}\Vert }_{2}}\right]$$

The overall training loss function was therefore defined as:

13 $${L}_{{\rm{total}}}={L}_{{\rm{rec}}}+{\lambda }_{{\rm{eff}}}{L}_{{\rm{eff}}}+{\lambda }_{{\rm{cor}}}{L}_{{\rm{cor}}}+{\lambda }_{{\rm{ref}}}{L}_{{\rm{ref}}}$$

where ${\lambda }_{{\rm{eff}}}$, ${\lambda }_{{\rm{cor}}}$, and ${\lambda }_{{\rm{ref}}}$ are weighting factors for each sub-term in Eq. (13). In accordance with the implementation, the correlation-based regularization terms were applied only during the early stages of training and were disabled after a predefined epoch threshold (e.g., 40 epochs), so that late-stage optimization focuses on maximizing display fidelity under the remaining constraints.

### Implementation details for numerical analysis

Numerical simulations of the diffractive 3D image display system were performed under coherent monochromatic illumination at a wavelength of *λ* = 0.75 mm. During the simulations, the input images were grayscale and resized to 32 × 32 pixels before being fed into the network, following the digital encoder and physical decoder described above. Each diffractive layer was composed of 256 × 256 trainable phase features (153.6*λ* × 153.6*λ*), with a lateral feature size of $d=0.6\lambda$. The central 32 × 32 region (19.2*λ* *×* 19.2*λ*) of the sensor plane was used for loss function calculation. In Fig. 6, we fixed the pixel pitch to $d=1\lambda$ to improve training stability for the larger SLM configuration, while keeping all the other settings the same.

The diffractive 3D display systems were trained using Python (v3.10.6) and JAX/Flax framework, utilizing the Optax library for optimization, with the Adam optimizer and a learning rate of $1\times {10}^{-4}$ for the digital encoder and$\,1\times {10}^{-3}$ for the diffractive decoder. The batch size was set to 64, and training was run for 2000 epochs. Diffractive layer phase values were initialized to zero. The training was performed on a GeForce RTX 4090 GPU (Nvidia Corp.), and each training run typically required ~24 h.

### Implementation details for experimental results

Experimental validation of the snapshot 3D image display system was performed using the optical setup illustrated in Fig. 8c. The system utilized a narrowband light source operating at a wavelength of *λ* = 650 nm, where the wavelength selection was provided by an integrated acousto-optic tunable filter (AOTF) module. The experimental system consisted of a digital encoder plane and a single diffractive decoder layer separated by an axial distance of *z* = 10 cm. The encoded phase patterns had 512 × 512 pixels, and the diffractive decoder had 900 × 900 pixels. The optical path was folded using two beam splitters (BS1 and BS2) to accommodate a two-stage modulation process involving two reflective SLMs (i.e., SLM1 and SLM2). The digital encoder first processed the target input images to generate the holographic phase pattern. This encoder pattern was displayed on SLM1 (HOLOEYE PLUTO-2.1; pixel pitch, 8 μm; resolution, 1920 × 1080), which served as the digital-to-optical interface, modulating the incident planar wavefront to create the encoded light field. The modulated field from SLM1 was directed by BS2 to SLM2 (HOLOEYE LUNA; pixel pitch, 4.5 μm; resolution, 1920 × 1080), which was positioned at an axial distance of *z* = 10 cm from SLM1. SLM2 acted as the physical decoder; it displayed the learned phase profile of the diffractive layer, effectively functioning as the passive, static diffractive surface described in the forward model. After modulation by the physical decoder, the light field propagated ~93 mm to the output region. A CMOS image sensor (Basler ace acA1920-40gm; pixel pitch, 5.86 μm; resolution, 1920 × 1200) was used to capture the resulting volumetric intensity distribution. A central 600 × 600 pixel region of the sensor was recorded and used for analysis. To evaluate the multi-plane display capability, the camera was mounted on a translation stage to record intensity measurements at multiple axial planes (e.g., 1st Plane and 2nd Plane as shown in Fig. 8a) corresponding to the depths of the target objects.

### Training and test datasets

We used the MNIST^48^ handwritten digits dataset for training and testing of most of the diffractive 3D display systems reported in this work. Specifically, 50k handwritten images were used for training, and the remaining 10k were reserved for testing. Individual digit images were randomly selected and assigned to different axial planes, and random combinations of digits were used to construct synthetic multi-plane targets for both training and testing. This dataset enables systematic evaluation of axial image routing, inter-plane cross-talk suppression, and generalization performance under well-controlled and widely adopted benchmarks. For the numerical simulations involving higher axial complexity, specifically the 28-slice volumetric image projection task, we used the 3D ShapeNets dataset^49^. Voxelized three-dimensional objects were represented as stacks of axial slices and used as depth-resolved supervision for training and blind testing. This dataset enabled the assessment of volumetric display performance for the presented system across a larger number of axial planes.

## Supplementary information

Supplementary Information

## Data Availability

All the data and methods needed to evaluate the conclusions of this work are presented in the main text and Supporting Information. Additional data can be requested from the corresponding author (A.O.).
