# Supplementary material for: Snapshot 3D image projection using a diffractive decoder
Source: Light Sci Appl. 2026 Jun 10;15:270. doi: 10.1038/s41377-026-02378-3 (PMC13250003; doi:10.1038/s41377-026-02378-3)
Supplement: Supplementary file 1 — Supplementary Information [file 41377_2026_2378_MOESM1_ESM.pdf]

Supplementary Information for
Snapshot 3D image projection using a diffractive decoder

Çağatay Işıl[1,2,3,†], Alexander Chen[1,†], Yuhang Li[1,2,3,†], F. Onuralp Ardic[1], Shiqi Chen[1,2,3,], Che-Yung Shen[1,2,3,], Aydogan Ozcan[1,2,3,*]

Affiliations

[1]Electrical and Computer Engineering Department, University of California, Los Angeles, CA, 90095, USA

[2]Bioengineering Department, University of California, Los Angeles, CA, 90095, USA

[3]California NanoSystems Institute (CNSI), University of California, Los Angeles, CA, 90095, USA

* ozcan@ucla.edu

[†] Equal contribution

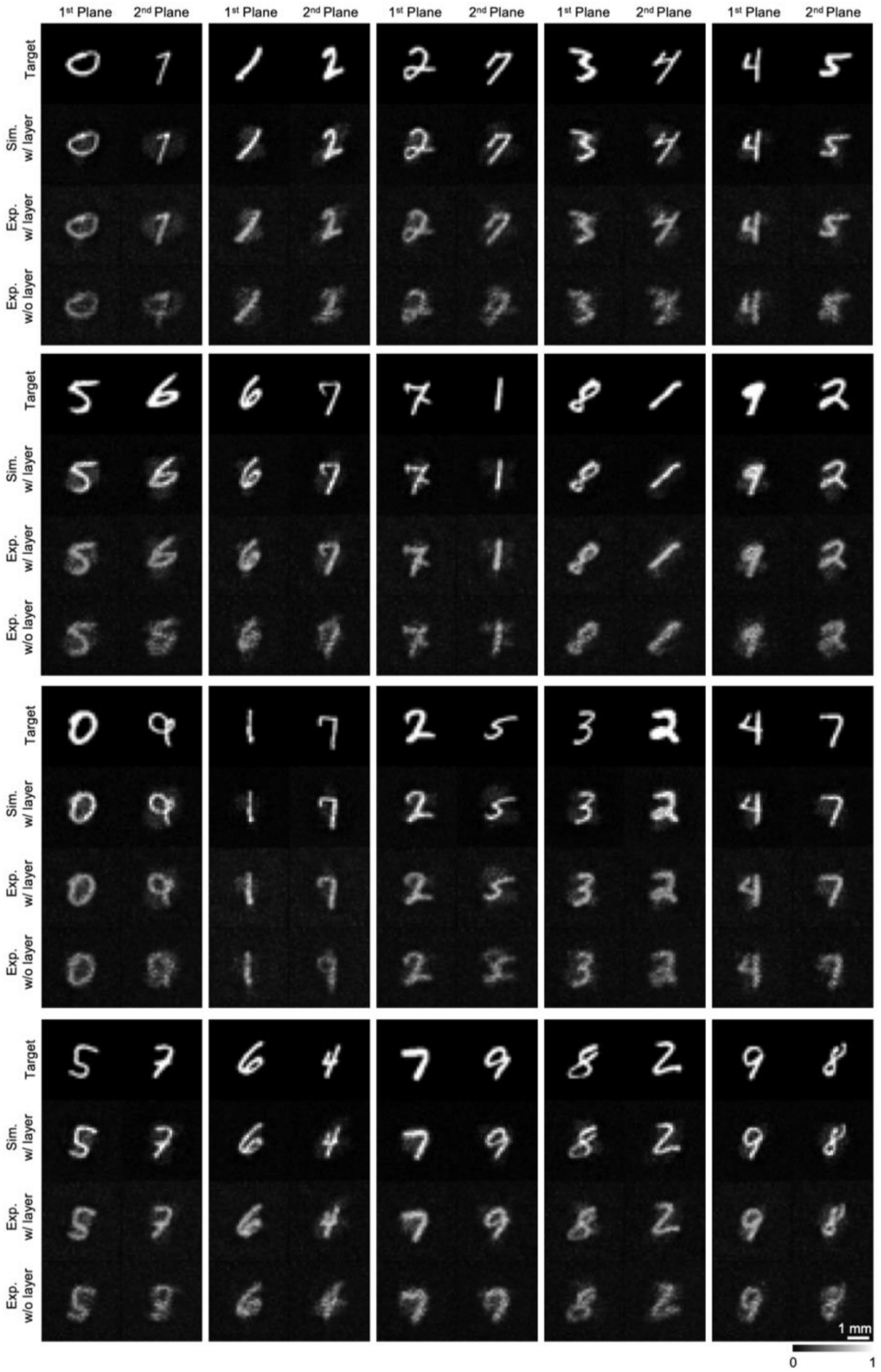

Supplementary Figure 1. Experimental validation of the diffractive snapshot 3D display system. Experimental results for multiple input test objects (never seen before). For each sample, target images (Target), simulated results with the diffractive layer (Sim. w/ layer), experimentally measured results with the diffractive layer (Exp. w/ layer), and experimentally measured free-space baseline without the diffractive layer (Exp. w/o layer) are shown at the 1st and 2nd image

planes. Also see Fig. 8 of the main text for additional experimental results that further support the same conclusions.
